# Endothelin-receptor antagonists are proapoptotic and antiproliferative in human colon cancer cells

**DOI:** 10.1038/sj.bjc.6600810

**Published:** 2003-03-04

**Authors:** L Peduto Eberl, R Bovey, L Juillerat-Jeanneret

**Affiliations:** 1 University Institute of Pathology, CHUV, University of Lausanne, Bugnon 25, CH1011 Lausanne, Switzerland

**Keywords:** endothelin, FasLigand, colon cancer, apoptosis, bosentan

## Abstract

Endothelin (ET)-1 can act as an autocrine/paracrine growth factor or an antiapoptotic factor in human cancers. To study the role of ET-1 in human colon cancer, proliferation and apoptosis of colon carcinoma cells was investigated using human HT-29 and SW480 colon carcinoma cells. ET-1 was secreted by these cells. Treatment of cells with bosentan, a dual ET_A/B_-receptor antagonist, decreased cell number. Inhibition of DNA synthesis by bosentan was observed only in the presence of serum. Exogenously added ET-1 did not increase DNA synthesis in serum-deprived cells. SW480 cells were sensitive and HT-29 cells were resistant to FasL-induced apoptosis. Bosentan sensitised resistant HT-29 cells to FasL-induced, caspase-mediated apoptosis, but not to TNF-*α*-induced apoptosis. Bosentan and/or FasLigand (FasL) did not modulate the expression of caspase-8 or FLIP. Bosentan sensitisation to apoptosis was reversed by low concentrations (10^−13^–10^−10^ M), but not by high concentrations (10^−9^–10^−7^ M) of ET-1. These results suggest that the binding of ET-1 to high-affinity sites inhibits FasL-induced apoptosis, while the binding of either ET-1 or receptor antagonists to low-affinity sites promotes FasL-induced apoptosis. In conclusion, endothelin signalling pathways do not induce human colon cancer cell proliferation, but are survival signals controling resistance to apoptosis.

Endothelins (ETs) 1, 2 and 3 are a family of 21 amino-acid peptides that mediate a variety of physiological functions, including cell growth and death. ETs are produced from inactive intermediates that are cleaved to yield active peptides by ET-converting enzyme (ECE), a zinc metalloprotease, specifically responsible for this key step (Shimada *et al*, 1995; Yanagisawa *et al*, 1998). The effects of ETs on mammalian cells are mediated by two distinct subtypes of G protein-coupled receptors, ET_A_ and ET_B_ (Arai *et al*, 1990; Sakurai *et al*, 1990), which are expressed in various tissues and stimulate several intracellular signalling systems. Several human cancer cell lines produce ET-1, with autocrine/paracrine growth factor functions (Kusuhara *et al*, 1990; Shichiri *et al*, 1991). Plasma ET-1 levels are elevated in patients with advanced colorectal cancer and ET-1 may be associated with metastatic progression (Shankar *et al*, 1998; Asham *et al*, 2001). Recently it has been suggested that ET-1, in addition to its mitogenic effects, may attenuate apoptosis. This novel role for ET-1 was demonstrated for rat endothelial cells (Shichiri *et al*, 1997), human smooth muscle cells (Wu-Wong *et al*, 1997), rat colon carcinoma cells (Peduto-Eberl *et al*, 2000) and human glioblastoma cells (Egidy *et al*, 2000c).

Proliferation and cell death must be properly balanced in order to maintain tissue homeostasis. This is achieved in part through mechanisms that interconnect the signalling pathways regulating these processes. Apoptosis is an active cell death process that takes place in a wide spectrum of physiological situations and is induced by several stimuli, including cytokines of the tumour necrosis factor (TNF) family. Interaction between the Fas receptor (CD95/APO-1), a member of the TNF-receptor family, and Fas ligand (FasL) triggers a pathway to cell death. However, resistance to cell death induced by the engagement of FasL on its receptor has been described in many cancers, involving various mechanisms, and suggesting that antiapoptotic pathways allow transformed cells to escape death. Susceptibility of a cell to apoptosis is also influenced by its state of proliferation and differentiation, depending on the particular cell type.

We have previously shown that the Fas/FasL and ET-1 systems are expressed in human colon carcinoma and in rat colon carcinoma cell lines (Peduto-Eberl *et al*, 1999, 2000b; Egidy *et al*, 2000a, 2000b). In human glioblastoma cells, blockade of the ET-1 pathway sensitised tumour cells to FasL-mediated apoptosis, decreasing the level of the short form of the FLICE/caspase-8-inhibitory protein (FLIP) in these cells (Egidy *et al*, 2000c). On the assumption that ET-1 might be also involved in resistance of human colon carcinoma cells to FasL-induced apoptosis, we studied the response of human colorectal cancer cell lines to ET-1 and ET-receptor antagonists.

## MATERIALS AND METHODS

### Immunohistochemistry

Human colon tissues were retrospectively selected from surgical colectomy specimens performed for cancer treatment. Immunohistochemical detection of ET-1 in paraffin-embedded human colon cancer tissue was performed essentially as previously described (Egidy *et al*, 2000a, 2000b, 2000c) using a monoclonal anti-ET-1 antibody (ABR, Alexis Corporation, Läufelingen, Switzerland).

### Detection of mRNAs by RT–PCR

Human colon carcinoma cell lines were from ATCC (American Tissue Type Collection, Manassas, VA, USA). Cells were grown in DMEM medium (Gibco-BRL, Basel, Switzerland) containing 4.5 g l^−1^ glucose, 10% fetal calf serum (FCS) and antibiotics. Total RNA from confluent cells was prepared using Trizol reagent (Gibco-BRL) and RT–PCR for the ET-1 system was performed according to standard procedures and specific primers as follows: PPET-1 (Egidy *et al*, 2000a, 341 bp; Pagotto *et al*, 1995, 304 bp); ECE-1 (Egidy *et al*, 2000a, 622 bp); ECE1_a,b,c,d_ (Egidy *et al*, 2000c, 353, 347, 348, 369 bp, respectively); ET_A_ (Egidy *et al*, 2000a, 675 bp, or (sense) 5′TTT GAT GTG GCA TTG AGC ATA CAG3′ (reverse) 5′CCT TTT GAT CAC AAT GAC TTT3′, 299 bp); ET_B_ (Egidy *et al*, 2000a, 400 bp, or (sense) 5′TTC CAA CGC CAG TCT GGC GCG GTC3′ (reverse) 5′GTC AAT ACT CAG AGC ACA TAG ACT3′, 425 bp); s-,l-FLIP (Egidy *et al*, 2000c, 507, 391 bp, respectively); Fas ((sense) 5′CAG AAC TTG GAA GGC CTG CAT C 3′ (reverse) 5′TCT GTT CTG CTG TGT CTT GGA C 3′, 682 bp); FasL ((sense) 5′CAA CTA TCT CGG TGC CTG TAA C3′ (reverse) 5′ CAG CTC TTC CAC CTA CAG AAG3′, 558 bp). To ensure the quality of RNAs, amplification reactions were performed with pairs of primers specific for human glyceraldehyde-phosphate dehydrogenase (Gapdh) (Egidy *et al*, 2000a, 456 bp).

### Measurement of ET secretion

HT-29 and SW480 cells were grown to confluence in DMEM containing 4.5 g l^−1^ glucose and 10% FCS, fresh medium with or without FCS was added and collected after 24, 48 or 72 h. The quantification of ET-1 in cell culture supernatants was performed using an enzyme-linked immunoassay (Biomedica, Wien, Austria) according to the supplier's instructions. Crossreactivity with big-ET-1, ET-2 and ET-3 was <1, 100 and <5%, respectively, according to the manufacturer's specification.

### Evaluation of ET-1 binding to cells

Cells were grown to confluence in the presence of FCS. The medium was changed to a new medium containing 10% FCS and 50 pM ^125^I-ET-1 (Anawa Trading, Wangen, Switzerland), and either 80 or 8 *μ*M bosentan or 3 nM ET-1 (Bachem, Bubendorf, Switzerland). After 1 h at room temperature, cells were washed, extracted in 1% SDS 0.1 N NaOH and supernatants and cell extracts counted in a *γ*-counter (Cobra, Packard, Perkin-Elmer, Hunenberg, Switzerland).

### Evaluation of cell growth

(3,4,5-Dimethylthiazol-yl)-2,5-diphenyl tetrazolium, (MTT) reduction was used to quantify metabolically active cells. Briefly, following treatment, cells were exposed to 0.25 mg ml^−1^ MTT (Sigma, Buchs, Switzerland) for 2 h. The cells were examined under a microscope to ascertain the density of violet spots corresponding to active mitochondria in order to exclude a potential mitochondrial toxicity of the compounds. The supernatant was then removed and the precipitated formazan was dissolved in 0.1 N HCl in isopropanol and quantified at 540 nm (iEMS, Labsystems, Bioconcepts, Allschwil, Switzerland).

Thymidine incorporation was used to assess cell proliferation. Cells were grown to 75% confluence, FCS was removed for 16–24 h, when indicated, and increasing concentrations of ET-1 or bosentan were added to the cells. At different times following agent addition, 1 *μ*Ci ml^−1^ [^3^H]thymidine (Amersham Pharmacia, Dübendorf, Switzerland) was added for 3–24 h and incorporation was quantitated in a *β*-counter (Rackbeta, LKB, Bertholel, Regensdorf, Switzerland) after precipitation with 10% trichloracetic acid and solubilisation in 0.1 N NaOH.

### FasL-induced cell death

The murine neuroblastoma cell line Neuro-2A FasL has been transfected to produce soluble murine FasL (Rensing-Ehl *et al*, 1995) and is used as a source of active FasL as previously described (Peduto-Eberl *et al*, 2000). Briefly, the collection of supernatants of Neuro-2A FasL cells was performed in a medium containing less than 0.5% FCS and the FasL titre of the preparations was controlled using murine 497 glioblastoma cells, which are responsive to FasL. Supernatant from *neo* vector Neuro-2A cells were used as control for FasL-containing supernatants. HT-29 and SW480 cells were maintained in DMEM 4.5 g l^−1^ glucose supplemented with 10% FCS. Cells were grown to half confluence, washed to eliminate any ET-1 in the culture medium for the initial period of exposure to effectors and incubated at 37°C with ET-receptor antagonists (bosentan (Actelion, Basel, Switzerland), BQ123 or BQ788 (both from RBI, Natick, MA, USA) at the indicated concentrations) in the presence or absence of FasL-containing supernatant and of 10% FCS for 24 or 48 h. For some experiments performed with bosentan, exogenous ET-1 was added at increasing concentrations from 10^−13^ to 10^−7^ M, together with FasL-containing supernatant for 24 h. For experiments performed with the caspase inhibitor zVAD-fmk (Bachem, Bubendorf, Switzerland), cells were preincubated with the inhibitor at the indicated concentrations and time, prior to the addition of bosentan and/or FasL-containing supernatant and incubation was continued for 24 h. As a control for the specificity of the FasL induction of apoptosis, cells were exposed to FasL-containing conditioned medium from Neuro 2A cells in the presence of 50 *μ*g ml^−1^ of the FasL- binding Fas-Fc-IgG fusion protein (Alexis Corporation, Taufaliugeu, Switzerland) to deplete specifically FasL from conditioned medium and bosentan. After 24 h, apoptosis was evaluated in adherent cells.

### Cell death evaluation

Apoptosis was quantified as previously described (Egidy *et al*, 2000c; Peduto-Eberl *et al*, 2000) using the Cell Death Detection ELISA^PLUS^ (Roche, Rotkreuz, Switzerland), a photometric enzyme-linked immunoassay for quantitative *in vitro* determination of cytoplasmic histone-associated DNA fragments (mono- and oligonucleosomes), according to the supplier's instructions. Increase in absorbance at 405 nm is proportional to apoptosis, and the enrichment in dead cell proportion (apoptosis index) was calculated as the ratio of absorbance of treated cells/absorbance of untreated cells.

### Flow cytometric analysis

HT-29 cells were detached by incubation with EDTA (1 mg ml^−1^) at 37°C for 10 min, essentially as previously described (Peduto-Eberl *et al*, 1999). After washing in PBS containing 2% FCS and incubation at 4°C for 30 min with the monoclonal mouse IgG1 anti-human Fas antibody (SM1/17) (Alexis Corporation, Ta"ufelingen, Switzerland), the cells were washed again and incubated with anti-mouse IgG-FITC (fluoroisothiocyanate)-conjugates (Alexis Corporation, Ta"ufelingen, Switzerland). After a further wash, cells were suspended in PBS containing 2% FCS and analysed on a FACScan flow cytometer (Becton-Dickinson and Co., San Jose, CA, USA). To label dead cells, 0.5 *μ*g ml^−1^ propidium iodide was added to the cells before analysis. Irrelevant monoclonal IgG antibodies (at equivalent concentrations) were used as isotype controls.

### Determination of p42/p44 MAP kinase/extracellular signal regulated kinase (ERK), FLIP and caspase-8 by Western blotting

Cells were grown in the presence of FCS, deprived of serum for 24 h and exposed or not to ET-1 for 10 min. Cell cultures were extracted using 0.1% Triton X-100 in the presence of a cocktail of protease inhibitors (Roche, Rotfzreuz, Switzerland) and submitted to SDS electrophoresis. Following transfer, the membrane was probed using monoclonal antiphosphorylated p42/p44 antibody (clone E10) (New England Biolabs, Bioconcepts, Alschwill, Switzerland) as previously described (Egidy *et al*, 2000c). Alternatively, cell extracts were exposed following blotting to anti-caspase-8 (New England Biolabs), anti-FLIP (a kind gift of J Tschopp, Lausanne, Switzerland) or anti *α*-SMA (Sigma, Buchs, Switzerland) monoclonal antibodies.

### Calculations of results

Each experiment was repeated at least three times unless otherwise stated. Means and s.d. were calculated. Statistical significance was assessed using an unpaired two-tailed Student's *t*-test.

## RESULTS

### Expression of the ET-1 system in human colon and colon carcinoma cells

**Figure 1:**
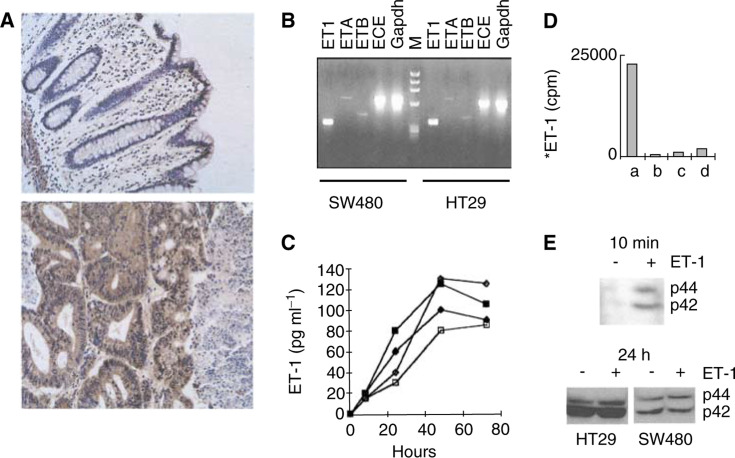
Imunnohistochemical localisation of ET-1 and expression of the mRNAs of the ET-1 pathway in human colon carcinoma cells. (**A**) ET-1 immunoreactivity is ubiquitously localised in tumour cells of colon carcinoma (lower panel), but not in nontumoral epithelial cells of normal colon (upper panel). (Original magnification: ×200). (**B**) HT29 and SW480 cells express the mRNAs of the ET system (PPET-1, ECE-1, ET_A_ and ET_B_). RT–PCR was performed on total RNA extracted from the colon carcinoma cell lines. Gapdh: glyceraldehyde-phosphate dehydrogenase. Two independent experiments gave similar results. (**C**) HT29 and SW480 cells secrete ET-1. HT-29 and SW480 cells were grown for 2 days in a culture medium containing 10% FCS, then half of the culture wells were deprived of serum and ET-1 was measured at various time intervals in the supernatants. ♦: HT-29 cells+FCS, ⋄: HT-29 cells no FCS; ▪: SW480 cells+FCS, □: SW480 cells no FCS. (**D**) SW480 cells bind ^125^I-ET-1 (50 pM), which is displaced by bosentan (8 or 80 *μ*M) or ET-1 (3 nM). Cells were grown in the presence of FCS for 3 days, and ET-1 binding was assessed in the presence of FCS (a: control; b: bosentan 80 *μ*M; c: bosentan 8 *μ*M; d: ET-1 3 nM). (**E**) ET-1 induces ERK phosphorylation in SW480 cells, indicating the presence of functional receptors. Cells were deprived of FCS for 24 h, then the medium was changed to a fresh medium without FCS either containing 0.4 nM ET-1 [+] or not containing ET-1 [−], and ERK phosphorylation was determined by Western blotting after 10 min exposure to ET-1. After 24 h in the absence of FCS without medium change, ERK is phosphorylated at a comparable extent in SW480 cells or HT-29 cells, exposed [+] or not exposed [−] to exogenous ET-1 (0.4 nM) for 10 min.

The presence of immunoreactive ET-1 was assessed in human normal colon (Figure 1A

, upper panel) and colon carcinoma (Figure 1A, lower panel). Cancer cells, but not normal epithelial cells, ubiquitously expressed ET-1. The expression of the mRNAs of the components of the ET system in HT-29 and SW480 human colon carcinoma cells was evaluated using RT–PCR. ET_A_ and ET_B_ receptors, ECE-1 and PPET-1 mRNAs confirmed the presence of all the transcripts of the ET system in these cells (Figure 1B).

Confluent cultures of SW480 (4×10^4^ cells cm^−2^) and HT-29 (5×10^4^ cells cm^−2^) cells secreted ET-1, either in the absence or presence of FCS (Figure 1C). The presence of cell membrane and functional ET-1 receptors in SW480 and HT-29 cells was evaluated using ^125^I-ET-1 binding (Figure 1D) and the phosphorylation of ERK, a known intracellular mediator of ET-1 (Figure 1E). SW480 cells, but not HT-29 cells, could bind ET-1 at concentrations corresponding to equilibrium constants of the receptors (Figure 1D). In SW480 cells exposed to fresh culture medium, phosphorylated-ERK was detected 10 min after addition of ET-1 (Figure 1E, 10 min), while both in SW480 cells and HT-29 cells, after 24 h culture without medium change, phosphorylation of ERK was detected and was not further increased by addition of ET-1 (Figure 1E, 24 h).

### ET-1 is not a proliferation-inducing factor in human colon carcinoma cells

**Figure 2:**
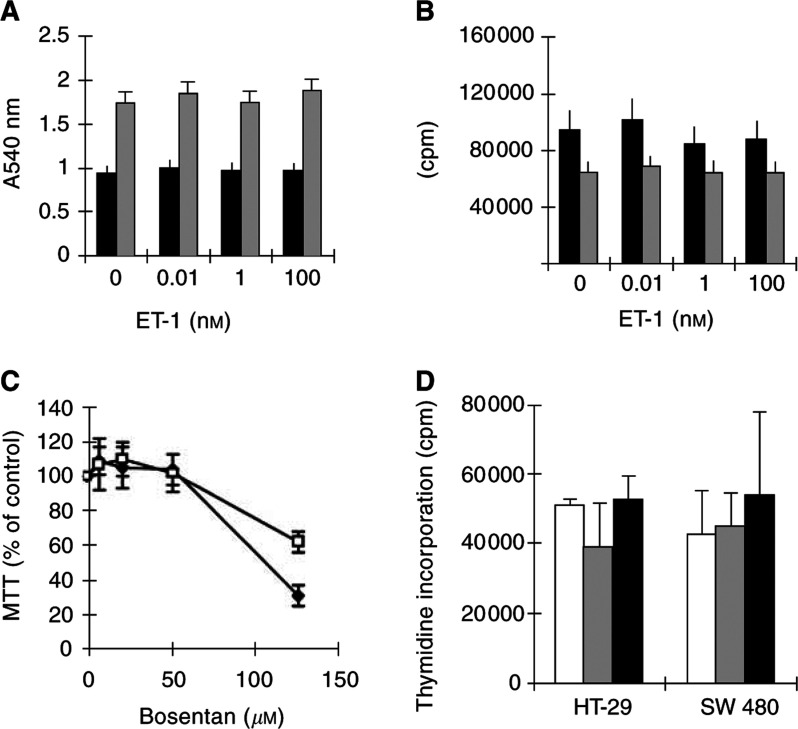
Role of bosentan and ET-1 in HT-29 and SW480 serum-deprived cells. Cells were grown to half-confluence, deprived of FCS, then bosentan or ET-1 were added for 24 h. Either MTT assay was performed or [^3^H]thymidine was added for the last 3 h of incubation to quantitate alive cell number or DNA synthesis, respectively. (**A**) ET-1 does not increase the number of metabolically active cells quantified using an MTT assay. (**B**) ET-1 does not induce DNA synthesis in HT-29 and SW480 cells. Grey bars: HT-29 cells, black bars: SW480 cells. (**C**) Bosentan decreases metabolically active cell number quantified using an MTT assay, HT-29 cells (♦), SW480 cells (□). (**D**) Bosentan does not inhibit DNA synthesis. White bars: no bosentan, grey bars: 10 *μ*M bosentan; black bars: 80 *μ*M bosentan. Experiments were repeated three times with identical information.

In serum-deprived colon carcinoma cells, ET-1 did not induce cell proliferation as determined either by MTT reduction, labelling metabolically active cells (Figure 2A

) or DNA synthesis, as determined by thymidine incorporation (Figure 2B). Blockade of ET_A_ and ET_B_ receptors by bosentan dose-dependently decreased the amount of metabolically active HT-29 and SW480 cells (Figure 2C). However, bosentan did not decrease DNA synthesis (Figure 2D).

**Figure 3:**
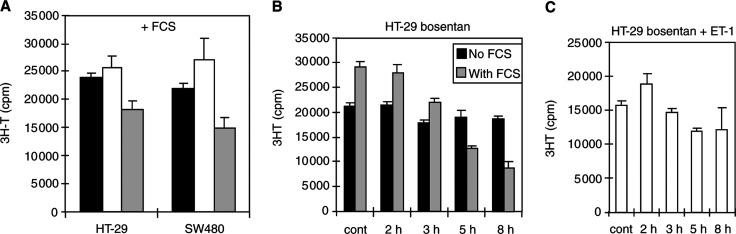
Bosentan inhibits plasma-induced DNA synthesis in colon carcinoma cells. HT-29 or SW480 cells were grown with 10% FCS, then exposed to bosentan in the absence of FCS for 2–9 h. [^3^H]Thymidine was added for the last 2 h of incubation. (**A**) Following culture in the presence of 10% FCS, 0 *μ*M (black bars), 8 *μ*M (white bars) or 80 *μ*M (grey bars) bosentan was added for 9 h. (**B**) Following 24 h culture of HT-29 cells in the presence of either 10% FCS (grey bars) or in the absence of FCS (black bars), either no bosentan ([cont], cultures after 8 h incubation in the absence of bosentan) or 80 *μ*M bosentan ([2, 3, 5, 8 h]) was added for 2, 3, 5 or 8 h. (**C**) In the presence of 40 nM ET-1 and 80 *μ*M bosnentan, no decrease in thymidine incorporation is observed for 2–8 h.

In serum-exposed cells, high (80 *μ*M), but not low (8 *μ*M), bosentan concentrations decreased DNA synthesis (Figure 3A

). In HT-29 cells (Figure 3B), bosentan time-dependently decreased DNA synthesis, which was abolished by addition of ET-1 conjointly to bosentan (Figure 3C). These results suggest that bosentan interfere with serum-derived factors in cells unable to bind ET-1 at concentrations corresponding to ET_A/B_-receptor equilibrium constants.

### Bosentan sensitises human colon carcinoma cell to FasL-induced apoptosis

**Figure 4:**
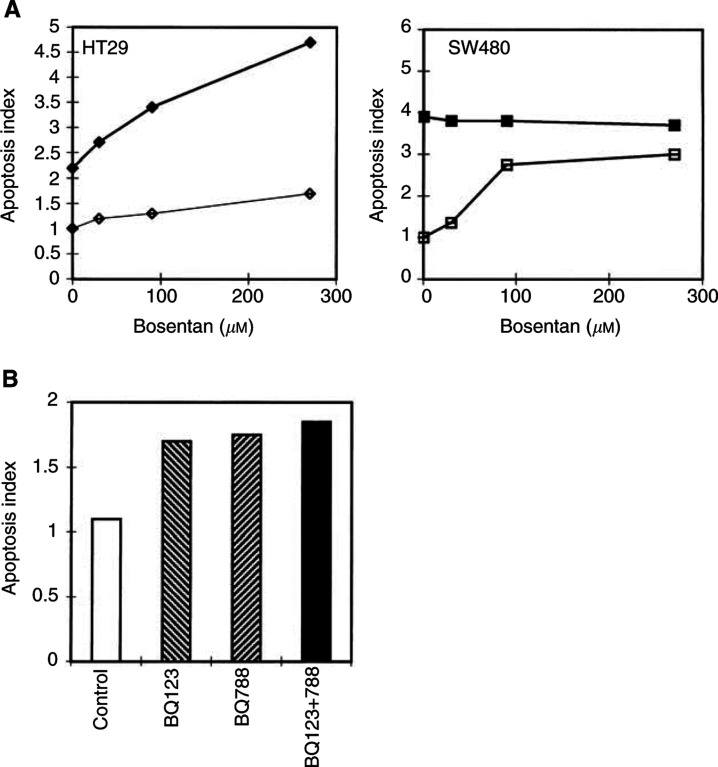
Bosentan sensitises FasL-resistant HT-29, but not FasL-sensitive SW480 carcinoma cells to FasL-induced apoptosis. (**A**) Half-confluent HT-29 (♦, ⋄) or SW480 (▪, □) cells were incubated with increasing concentrations of bosentan (10–270 *μ*M) in the absence (open symbols) or in the presence (closed symbols) of FasL-containing supernatant from neo-Neuro2A/FasL cells. Apoptosis was evaluated after 24 h of incubation. (**B**) Two ET-receptor antagonists structurally unrelated to bosentan, BQ123 (ET_A_-specific) and BQ788 (ET_B_-specific) significantly, but not synergistically, sensitise HT29 cells to FasL-mediated apoptosis. Half-confluent HT-29 cells were incubated in the presence of 80 *μ*M of either BQ123 or BQ788 or BQ123 and BQ788 in combination, and FasL-containing supernatant from neo-Neuro2A/FasL cells. (Apoptosis index=1 is defined as the apoptosis measured in the absence of all effectors). Apoptosis was evaluated after 24 h of incubation. Means±s.d. were calculated. One representative experiment out of four is shown.

Increasing concentrations of bosentan were added to half confluent cells and nucleosomal fragmentation was quantified. Bosentan alone dose-dependently increased apoptosis in SW480 cells, while it had little effect on HT29 cells (Figure 4

, open symbols). In the presence of FasL, HT29 cells were partially resistant to FasL-mediated apoptosis after 24 h of treatment, while SW480 cells were sensitive to FasL-induced apoptosis (Figure 4, closed symbols), confirming previous information for SW480 cells (Möller *et al*, 1994). Treatment of HT-29 cells with FasL and increasing concentrations of bosentan for 24 h significantly enhanced FasL-mediated apoptosis (Figure 4, closed symbols), while it had no enhancing effects on the sensitivity of SW480 cells to FasL. Comparable information was obtained using propidium iodide-labelled HT-29 cells and FACS analysis, as previously described for rat colon carcinoma cells (Peduto-Eberl *et al*, 2000) (results not shown). The addition of Neuro2A-control medium alone or in the presence of bosentan did not potentiate apoptosis and apoptosis was inhibited in cells exposed to FasL in the presence of the FasL-binding Fas-Fc-IgG fusion protein and bosentan (results not shown), confirming that apoptosis was because of Fas–FasL interactions. By RT–PCR, both cell lines expressed Fas mRNA, but only SW480 cells express FasL mRNA (not shown).

**Figure 5:**
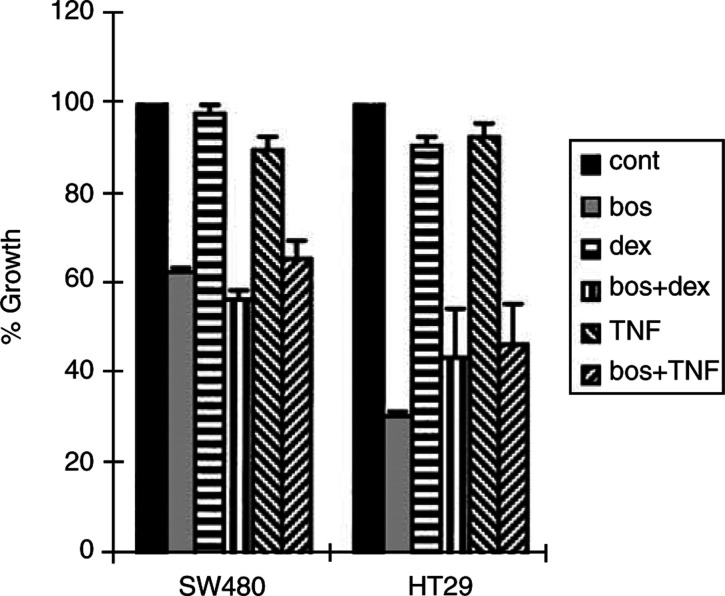
TNF-*α* or dexamethasone do not potentiate bosentan effects on growth of human colon carcinoma cells. Half-confluent HT-29 or SW480 cells were incubated with bosentan (bos) and/or TNF-*α* (TNF) or dexamethasone (dex). MTT assay was performed for the last 3 h of incubation to quantitate metabolically active cell number. Means±s.d. were calculated. One representative experiment out of two is shown.

Two ET-receptor antagonists structurally unrelated to bosentan, BQ123 (ET_A_-specific) and BQ788 (ET_B_-specific), were investigated. Both BQ123 or BQ788 (at 80 *μ*M concentrations) alone or in combination significantly (Figure 4C; *P*<0.0004 for all treatments *vs* control), but not synergistically, sensitised HT29 cells to FasL-mediated apoptosis like bosentan. This information suggests that both ET_A_ and ET_B_ receptors can transmit the antiapoptotic signal. Other apoptosis-inducing factors, the glucocorticoid dexamethasone (10 *μ*g ml^−1^) or TNF-*α* (100 U ml^−1^) (Figure 5

), did not potentiate the effects of bosentan on decreasing the number of metabollically active cells, indicating that bosentan sensitisation to apoptosis is specific to FasL-induced apoptosis in these cells.

**Figure 6:**
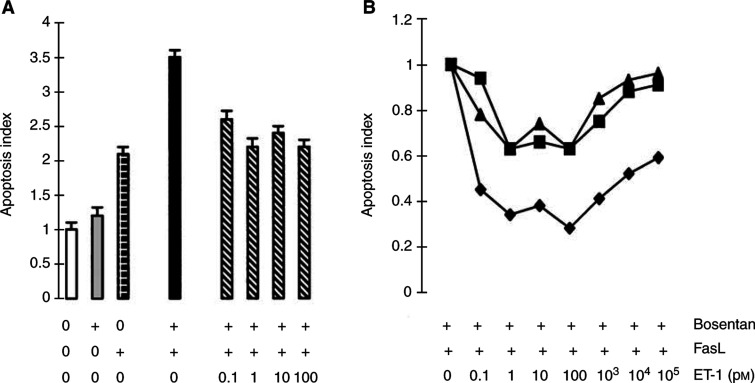
Low concentrations of exogenous ET-1 antagonises and high concentrations of ET-1 promotes bosentan-induced apoptosis. (**A**) Low concentrations of exogenous ET-1 antagonises bosentan-induced apoptosis (apoptosis index=1 in the absence of bosentan, FasL and ET-1) in HT-29 cells. Half-confluent human HT-29 colon carcinoma cells were incubated simultaneously with bosentan (80 *μ*M), FasL and increasing concentrations of ET-1 (10^−13^–10^−10^ M). Apoptosis was evaluated after 24 h of incubation. (**B**) High concentrations of ET-1 promotes apoptosis (apoptosis index=1 in the presence of bosentan and FasL, without ET-1). Half-confluent human HT-29 (▪) or rat PROb (▴) and REGb (♦) colon carcinoma cells were incubated simultaneously with bosentan (80 *μ*M for HT-29 cells; or 25 *μ*M for PROb and REGb cells), FasL and increasing concentrations of ET-1 (10^−13^–10^−7^ M). Apoptosis was evaluated after 24 h of incubation. Means+s.d. were calculated. One representative experiment out of three is shown.

Addition of low concentrations of exogenous ET-1 (10^−13^–10^−10^ M) to HT-29 cells together with 80 *μ*M bosentan and FasL antagonised bosentan sensitisation to FasL-induced apoptosis (Figure 6A

), confirming that bosentan enhancement of FasL-induced apoptosis was dependent on the ET-1 pathway. However, at higher concentration of ET-1 (10^−9^–10^−7^ M), the antagonistic effect of ET-1 was no longer observed, indicating that at high concentrations ET-1 promoted apoptosis. Similar observation was obtained using the FasL-resistant, bosentan-sensitive (Peduto-Eberl *et al*, 2000) rat colon carcinoma PROb and REGb cells (Figure 6B).

### Evaluation of the caspase-8 pathway in bosentan-mediated effects in colon carcinoma cells

**Figure 7:**
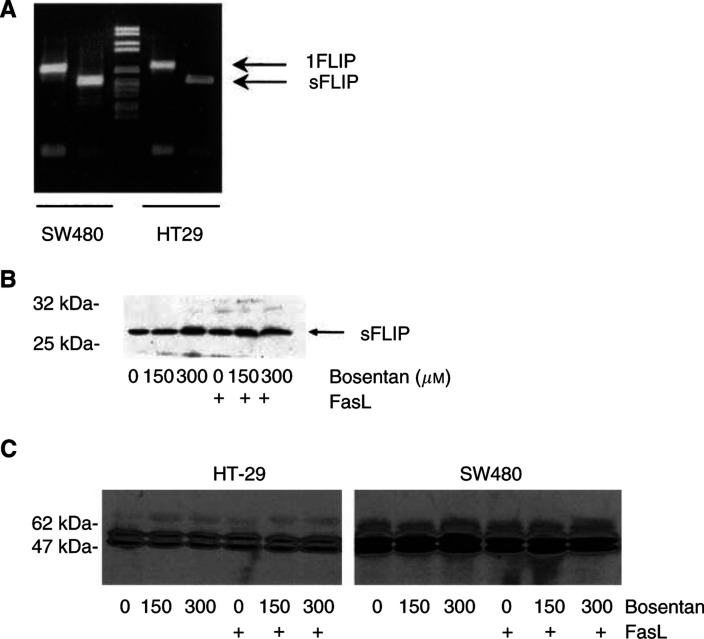
Expression of the FLIP and caspase-8 in HT29 and SW480 cells. (**A**) HT-29 and SW480 cells express the long and the short FLIP mRNAs. RT–PCR was performed on total RNA extracted from the two tumoral cell lines. (**B**) Bosentan and FasL do not modulate the expression of the short form of FLIP in SW480 cells. Half-confluent SW480 cells were incubated with bosentan and/or FasL for 24 h and the presence of sFLIP was determined by Western blotting. (**C**) Bosentan and FasL do not modulate the expression of caspase-8 in HT-29 and SW480 cells exposed to bosentan and FasL. Half-confluent SW480 or HT-29 cells were incubated with bosentan and/or FasL for 24 h and the presence of caspase-8 was determined by Western blotting. Two independent experiments gave similar results.

**Figure 8:**
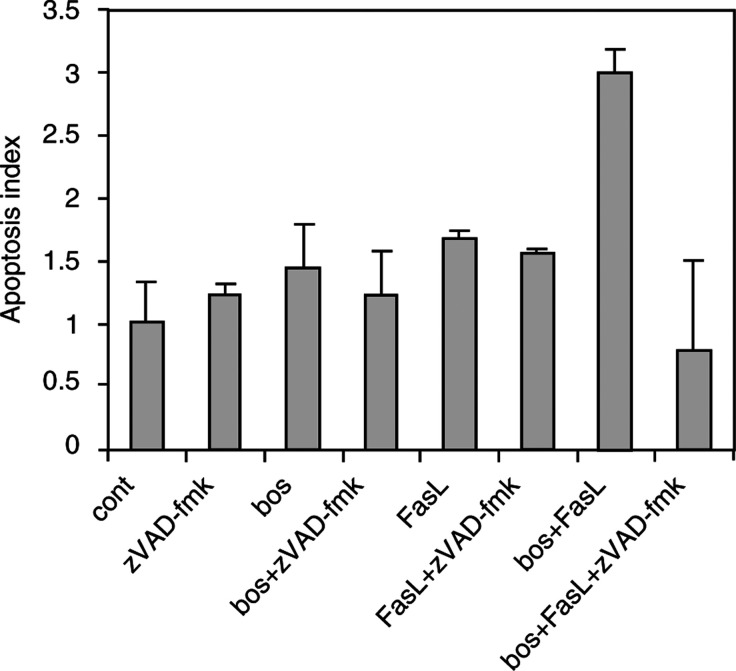
Bosentan sensitisation to FasL-induced apoptosis in HT-29 cells is blocked by the general caspase inhibitor zVAD-fmk. Cells were preincubated with 100 *μ*M zVAD-fmk for 1 h, then 150 *μ*M bosentan (without FasL) or 80 *μ*M bosentan (with FasL) were added. Incubation was continued for 24 h and apoptosis was evaluated. Values are the mean+s.d. of two independent determinations. *P*<0.01: bosentan+FasL *vs* control; z-VAD-fmk+bosentan+FasL *vs* bosentan+FasL.

To study the mechanism of bosentan sensitisation to FasL-mediated apoptosis, the expression of Fas/FasL, FLIP and the involvement of caspase activity were evaluated after bosentan and/or FasL treatment. By flow cytometry, Fas was expressed at low levels in HT29 cells, and its expression was not increased by exposure to bosentan (results not shown) suggesting that ET-1 does not interfere with the Fas pathway at the receptor expression level, in contrats with IFN-*γ*-dependent induction of Fas in these cells (Möller *et al*, 1994). Both cell lines expressed mRNAs for the long and short form of FLIP (Figure 7A

) and the long form of FLIP protein (not shown); however, only SW480 cells expressed detectable levels of the short form of FLIP protein (Figure 7B). Exposure of SW480 cells to bosenatn and/or FasL neither decreased the level of the short form of FLIP as previously shown for glioblastoma cells (Egidy *et al*, 2000c) (Figure 7B), nor modify caspase-8 expression (Figure 7C). The long form of FLIP was not modified in both cell lines by these treatments (not shown). Involvement of caspase activity in FasL/bosentan-dependent apoptosis in HT29 cells was therefore demonstrated: pre- and coincubation of cells with 100 *μ*M of the general caspase inhibitor zVAD-fmk in the presence of both bosentan and FasL in HT-29 cells inhibited apoptosis (Figure 8

).

## DISCUSSION

Tumour progression is dependent upon equilibrium between cell death-promoting and growth-promoting factors. Death-promoting factors include FasL, while ET-1 is considered as a growth-promoting factor. We have previously shown that both the complete ET-1 (Egidy *et al*, 2000a, 2000b) and Fas/FasL (Peduto-Eberl *et al*, 1999) systems are expressed in human normal colon and colon carcinoma. We demonstrate here that ET-1 immunoreactivity is highly expressed in human colon cancer and that human colon carcinoma cells secrete ET-1. Several human cancer cell lines have been shown to produce ET-1 with autocrine/paracrine growth factor functions (Kusuhara *et al*, 1990; Shichiri *et al*, 1991), and ET-1 has been implicated in metastasis of colon cancer (Shankar *et al*, 1998; Asham *et al*, 2001) and as an apoptosis survival factor in endothelial cells (Shichiri *et al*, 1997), smooth muscle cells (Wu-Wong *et al*, 1997) and fibroblasts (Shichiri *et al*, 1998). Resistance to FasL-induced apoptosis of rat colon carcinoma cells involved the ET-1 system and in a syngenic rat model of carcinomatosis, bosentan treatment resulted in a trend towards a lower tumour grading (Peduto-Eberl *et al*, 2000). Thus in the present approach, we investigated the effects of blockade of the ET-1 system in human colon cancer cells.

The human colon carcinoma HT-29 and SW480 cells expressed all the components of the ET-1 and Fas/FasL systems and secreted ET-1, thus representing good models to study the role of ET-1 in colon cancer. Exogenously added ET-1 was not directly involved in the induction of cell proliferation. However, activation of the ET-1 pathway was a necessary permissive signal for colon carcinoma cell survival. This information is in agreement with our previous observation that in induced colon carcinomatosis in the rat, bosentan, a dual ET_A_/ET_B_-receptor antagonist (Clozel *et al*, 1993), has the potential to reduce initial tumour growth (Peduto-Eberl *et al*, 2000; Egidy *et al*, 2000). In human colon carcinoma cells, bosentan induced low levels of apoptosis in SW480 cells and potentiated FasL-mediated apoptosis in FasL-resistant HT-29 cells. In our experiments, exposure to bosentan did not significantly modify Fas, FLIP or caspase-8 expression, which suggests that the ET-1 pathways does not directly interfere with expression of the molecules of the Fas pathway in the control of apoptosis in these cells. This information also suggests that antiapoptotic molecules other than FLIP or different intracellular regulatory pathways are involved in carcinoma cells when compared to glioblastoma cells, as we had previously shown (Egidy *et al*, 2000c) that in human glioblastoma cells, bosentan could decrease the levels of the short form of the FLIP protein. However, in human colon cancer as in glioblastoma cells (Egidy *et al*, 2000c), ET-1 is not a proliferation-inducing factor, but is necessary for the survival of cancer cells.

In our experiments, low concentrations of ET-1 (10^−13^–10^−10^ M) antagonised bosentan-induced apoptosis in HT-29 cells, even in the presence of a high concentration (80 *μ*M) of bosentan. These low concentrations of ET-1 are comparable to ET-1 plasma levels and to the levels secreted by colon carcinoma cells. Thus ET-1 is not a proliferation-inducing factor for human colon carcinoma cells; however, ET-1 is necessary for tumour cell survival. At high ET-1 concentrations, ET-1 did not stimulate DNA synthesis but sensitised HT-29 death-resistant cells to FasL/bosentan-induced apoptosis. Thus, at physiological plasma concentrations, ET-1 may exert an antiapoptotic effect, while at high concentrations ET-1 and bosentan are proapototic. Therefore, ET-1 production by colon cell lines is sufficient for this peptide to act as an autocrine survival factor, but not a proapoptotic factor. Interestingly, exogenous radioactive ET-1, at concentrations corresponding to the affinity constants of this peptide for its receptors, was bound only by SW480 cells, not by HT-29 cells. These results suggest that ET-receptor antagonists have binding sites different from the cell-surface ET_A/B_ receptors, and also suggest that ET peptides and antagonists, including bosentan, BQ123 or BQ788, have two binding sites in human colon cancer cells: a high-affinity binding site, whose occupancy by ET-1 protects against FasL-induced apoptosis, and a low-affinity binding site, whose occupancy either by ET-1 or receptor antagonists sensitises cells to apoptosis and whose exact nature is presently not defined. Alternatively, the effects of ET-receptors antagonists involve intracellular targets, and the high concentrations of antagonists necessary to observe an effect depends on delivery of the molecules into the cells.

Upon interaction of Fas, a member of the TNF family, with its ligand, FasL, death is induced via caspase activation. Several mechanisms may render cells resistant to FasL-induced apoptosis. Bosentan sensitisation to FasL-mediated apoptosis in HT29 cells was completely blocked by the general caspase inhibitor zVAD-fmk, demonstrating the involvement of the caspase proteases. These results suggest that ET-1-receptor blockade allows HT-29 cells to undergo caspase activation, via the Fas pathway. However, ET-1 does not sensitise cells to death induced by TNF-*α*, another member of the TNF-death receptor family.

In conclusion, we have shown that ET-1 is not a proliferation-inducing factor in human colon carcinoma. Blockade of ET receptors either induces apoptosis or sensitises cells to Fas-induced apoptosis. In colon cancer cells, low concentrations of ET-1, either added exogenously or secreted by the tumour cells, are permissive for colon cancer cell survival, promoting resistance to FasL-mediated apoptosis, while high concentrations of either receptor antagonists or ET-1 promote apoptosis.
